# Supplementary material for: The role of ambient temperature and body mass on body temperature, standard metabolic rate and evaporative water loss in southern African anurans of different habitat specialisation
Source: PeerJ. 2019 Oct 22;7:e7885. doi: 10.7717/peerj.7885 (PMC6814148; doi:10.7717/peerj.7885)
Supplement: Supplemental Information 1 [file peerj-07-7885-s001.docx]

**Supplementary information**

Supplementary table S1. Comparison of variable importance (R2m and R2c) between Vapour Pressure Deficit (VPD) vs Temperature in driving rates of Evaporative Water Loss (EWL) in the three test species. VPD was a better predictor for EWL in both *A. delalandii* and *S. capensis*, but not in *X. laevis*.

| Species |  | Covariate |  | *R^2^_(m)_* | *R^2^_(c)_* |
| --- | --- | --- | --- | --- | --- |
| *Amietia delalandii* |  | VPD |  | 0.313 | 0.431 |
|  |  | Temperature |  | 0.266 | 0.352 |
| *Sclerophrys capensis* |  | VPD |  | 0.223 | 0.223 |
|  |  | Temperature |  | 0.210 | 0.210 |
| *Xenopus laevis* |  | VPD |  | 0.437 | 0.437 |
|  |  | Temperature |  | 0.445 | 0.445 |

Supplementary table S2*.* Generalised linear mixed-model results for *Xenopus laevis* including *R*^2^ and semi-partial *R*^2^ estimates for fixed variables.

|  |  |  | |  | | ***R*^2^ estimates** | | |
| --- | --- | --- | --- | --- | --- | --- | --- | --- |
| **Variable** | **Fixed effect** | **Estimate** | **SE** | | ***p*-value** | **Semi- partial** | ***R*^2^ _(_*_c_*_)_** | ***R*^2^ *_(m)_*** |
| Body temperature (*T*_b_) | Intercept | 7.654 | 0.529 | | <0.0001 | 0.987 | 0.993 | 0.987 |
|  | Temp 10 | 3.106 | 0.386 | | <0.0001 | 0.611 |  |  |
|  | Temp 15 | 7.506 | 0.365 | | <0.0001 | 0.894 |  |  |
|  | Temp 20 | 12.059 | 0.383 | | <0.0001 | 0.953 |  |  |
|  | Temp 25 | 15.531 | 0.377 | | <0.0001 | 0.957 |  |  |
|  | Temp 30 | 19.253 | 0.282 | | <0.0001 | 0.971 |  |  |
|  | Temp 35 | 22.646 | 0.006 | | < 0.0001 | 0.980 |  |  |
|  | Sex | -0.404 | 0.357 | | 0.283 | 0.163 |  |  |
|  | Mass | 0.002 | 0.006 | | 0.628 | 0.105 |  |  |
| Evaporative water loss (EWL) | Intercept | 0.249 | 0.036 | | <0.001 | 0.614 | 0.699 | 0.611 |
|  | Temp 10 | -0.033 | 0.028 | | 0.230 | 0.021 |  |  |
|  | Temp 15 | 0.023 | 0.027 | | 0.520 | 0.011 |  |  |
|  | Temp 20 | -0.034 | 0.028 | | 0.092 | 0.023 |  |  |
|  | Temp 25 | 0.122 | 0.028 | | <0.05 | 0.232 |  |  |
|  | Temp 30 | 0.123 | 0.029 | | <0.05 | 0.233 |  |  |
|  | Temp 35 | 0.137 | 0.027 | | <0.05 | 0.285 |  |  |
|  | Sex | -0.025 | 0.023 | | 0.294 | 0.020 |  |  |
|  | Mass | 0.000 | 0.000 | | 0.680 | 0.004 |  |  |
| Standard metabolic rate (SMR) | Intercept | 7.630 | 1.075 | | <0.0001 | 0.539 | 0.560 | 0.538 |
|  | Temp 10 | -0.795 | 0.907 | | 0.390 | 0.013 |  |  |
|  | Temp 15 | 0.461 | 0.876 | | 0.604 | 0.005 |  |  |
|  | Temp 20 | -0.826 | 0.899 | | 0.367 | 0.014 |  |  |
|  | Temp 25 | 3.350 | 0.896 | | <0.001 | 0.195 |  |  |
|  | Temp 30 | 3.297 | 0.904 | | <0.001 | 0.189 |  |  |
|  | Temp 35 | 3.577 | 0.873 | | <0.0001 | 0.224 |  |  |
|  | Sex | -0.854 | 0.672 | | 0.217 | 0.029 |  |  |
|  | Mass | 0.000 | 0.011 | | 0.934 | 0.000 |  |  |

***R*^2^ _(_*_c_*_)_ =** conditional *R*^2^; ***R*^2^ _(_*_c_*_)_ =** marginal *R*^2^**;** SE = Standard error; Temp = Temperature

Supplementary table S3*.* Generalised linear mixed-model results for *Amietia delalandii* including *R*^2^ and semi-partial *R*^2^ estimates for fixed variables.

|  |  |  | |  | | **R^2^ estimates** | | |
| --- | --- | --- | --- | --- | --- | --- | --- | --- |
| **Variable** | **Fixed effect** | **Estimate** | **SE** | | ***p*-value** | **Semi- partial** | **R^2^ _(_*_c_*_)_** | **R^2^ *_(m)_*** |
| Body temperature (*T*_b_) | Intercept | 5.070 | 0.414 | | <0.0001 | 0.992 | 0.994 | 0.992 |
|  | Temp 10 | 4.126 | 0.330 | | <0.0001 | 0.720 |  |  |
|  | Temp 15 | 8.446 | 0.342 | | <0.0001 | 0.908 |  |  |
|  | Temp 20 | 12.774 | 0.328 | | <0.0001 | 0.960 |  |  |
|  | Temp 25 | 16.423 | 0.334 | | <0.0001 | 0.975 |  |  |
|  | Temp 30 | 20.929 | 0.353 | | <0.0001 | 0.983 |  |  |
|  | Temp 35 | 23.415 | 0.333 | | <0.0001 | 0.992 |  |  |
|  | Sex | 0.269 | 0.262 | | 0.320 | 0.023 |  |  |
|  | Mass | 0.045 | 0.016 | | <0.01 | 0.160 |  |  |
| Evaporative water loss (EWL) | Intercept | 0.216 | 0.036 | | <0.0001 | 0.388 | 0.568 | 0.388 |
|  | Temp 10 | 0.017 | 0.028 | | 0.553 | 0.006 |  |  |
|  | Temp 15 | 0.026 | 0.029 | | 0.37 | 0.013 |  |  |
|  | Temp 20 | -0.020 | 0.027 | | 0.477 | 0.008 |  |  |
|  | Temp 25 | 0.047 | 0.028 | | 0.107 | 0.041 |  |  |
|  | Temp 30 | 0.077 | 0.030 | | <0.01 | 0.097 |  |  |
|  | Temp 35 | 0.130 | 0.028 | | <0.0001 | 0.253 |  |  |
|  | Sex | 0.008 | 0.023 | | 0.743 | 0.003 |  |  |
|  | Mass | 0.002 | 0.001 | | 0.258 | 0.031 |  |  |
| Standard metabolic rate (SMR) | Intercept | 6.011 | 1.045 | | <0.0001 | 0.370 | 0.558 | 0.558 |
|  | Temp 10 | 0.262 | 0.815 | | 0.751 | 0.002 |  |  |
|  | Temp 15 | 0.808 | 0.845 | | 0.348 | 0.014 |  |  |
|  | Temp 20 | -0.594 | 0.809 | | 0.470 | 0.008 |  |  |
|  | Temp 25 | 1.284 | 0.823 | | 0.133 | 0.036 |  |  |
|  | Temp 30 | 2.114 | 0.874 | | <0.05 | 0.086 |  |  |
|  | Temp 35 | 3.518 | 0.824 | | <0.001 | 0.221 |  |  |
|  | Sex | 0.238 | 0.667 | | 0.724 | 0.003 |  |  |
|  | Mass | 0.062 | 0.041 | | 0.137 | 0.054 |  |  |

***R*^2^ _(_*_c_*_)_ =** conditional *R*^2^; ***R*^2^ _(_*_c_*_)_ =** marginal *R*^2^**;** SE = Standard error; Temp = Temperature

Supplementary table S4*.* Generalised linear mixed-model results for *Sclerophrys capensis* including *R*^2^ and semi-partial *R*^2^ estimates for fixed variables.

|  |  |  | |  | | **R^2^ estimates** | | |
| --- | --- | --- | --- | --- | --- | --- | --- | --- |
| **Variable** | **Fixed effect** | **Estimate** | **SE** | | ***p*-value** | **Semi- partial** | **R^2^ _(_*_c_*_)_** | **R^2^ *_(m)_*** |
| Body temperature (*T*_b_) | Intercept | 5.354 | 0.751 | | <0.0001 | 0.979 | 0.979 | 0.979 |
|  | Temp 10 | 4.724 | 0.573 | | <0.0001 | 0.553 |  |  |
|  | Temp 15 | 8.546 | 0.586 | | <0.0001 | 0.795 |  |  |
|  | Temp 20 | 13.371 | 0.590 | | <0.0001 | 0.889 |  |  |
|  | Temp 25 | 17.694 | 0.575 | | <0.0001 | 0.945 |  |  |
|  | Temp 30 | 20.435 | 0.569 | | <0.0001 | 0.959 |  |  |
|  | Temp 35 | 21.422 | 0.573 | | <0.0001 | 0.962 |  |  |
|  | Sex | 0.515 | 0.182 | | 0.169 | 0.036 |  |  |
|  | Mass | 0.018 | 0.008 | | <0.05 | 0.099 |  |  |
| Evaporative water loss (EWL) | Intercept | 0.143 | 0.047 | | <0.001 | 0.634 | 0.634 | 0.634 |
|  | Temp 10 | -0.043 | 0.035 | | 0.240 | 0.026 |  |  |
|  | Temp 15 | -0.085 | 0.036 | | <0.05 | 0.090 |  |  |
|  | Temp 20 | 0.094 | 0.037 | | <0.05 | 0.107 |  |  |
|  | Temp 25 | -0.017 | 0.036 | | 0.646 | 0.004 |  |  |
|  | Temp 30 | 0.187 | 0.035 | | <0.0001 | 0.336 |  |  |
|  | Temp 35 | 0.130 | 0.036 | | <0.001 | 0.195 |  |  |
|  | Sex | 0.044 | 0.022 | | 0.066 | 0.065 |  |  |
|  | Mass | 0.001 | 0.000 | | 0.064 | 0.063 |  |  |
| Standard metabolic rate (SMR) | Intercept | 4.720 | 1.464 | | <0.001 | 0.578 | 0.578 | 0.578 |
|  | Temp 10 | 0.085 | 1.116 | | 0.940 | 0.000 |  |  |
|  | Temp 15 | -2.982 | 1.142 | | 0.883 | 0.110 |  |  |
|  | Temp 20 | 1.434 | 1.149 | | <0.05 | 0.028 |  |  |
|  | Temp 25 | -1.331 | 1.121 | | 0.245 | 0.025 |  |  |
|  | Temp 30 | 4.366 | 1.110 | | <0.001 | 0.220 |  |  |
|  | Temp 35 | 3.105 | 1.118 | | <0.05 | 0.123 |  |  |
|  | Sex | 1.137 | 0.702 | | 0.136 | 0.045 |  |  |
|  | Mass | 0.034 | 0.015 | | <0.05 | 0.086 |  |  |

***R*^2^ _(_*_c_*_)_ =** conditional *R*^2^; ***R*^2^ _(_*_c_*_)_ =** marginal *R*^2^**;** SE = Standard error; Temp = Temperature; VPD = Vapour pressure deficit.
